# Ultra-Processed Food Consumption Among Caregivers and Children in the “Happy Smile” Project: Associations with Family Dietary Patterns and Periodontal Health-Related Quality of Life

**DOI:** 10.3390/nu18040678

**Published:** 2026-02-19

**Authors:** Vitor Hugo Gonçalves Sampaio, Guilherme Assumpção Silva, Amanda Rodrigues Araújo, Ana Laura Gavaldão Santana Moreira, Letícia Helena Theodoro, Alessandra Marcondes Aranega, Cristina Antoniali Silva, Daniela Atili Brandini

**Affiliations:** 1 Department of Diagnosis and Surgery, School of Dentistry of Araçatuba, São Paulo State University, Araçatuba 16015-050, SP, Brazil; vitor.sampaio@unesp.br (V.H.G.S.); guilherme.assumpcao@unesp.br (G.A.S.); amanda.rodrigues-araujo@unesp.br (A.R.A.); ana.laura2004@unesp.br (A.L.G.S.M.); leticia.theodoro@unesp.br (L.H.T.); alessandra.aranega@unesp.br (A.M.A.); 2 Department of Basic Sciences, School of Dentistry of Araçatuba, São Paulo State University, Araçatuba 16015-050, SP, Brazil; cristina.antoniali@unesp.br

**Keywords:** ultra-processed foods, quality of life, oral health, preschool child, caregivers

## Abstract

**Background/Objectives:** The consumption of ultra-processed foods (UPFs) has increased markedly in recent decades and has been associated with adverse health outcomes. In childhood, the family environment plays a central role in shaping dietary habits and oral health behaviors. This study investigated the association between UPF consumption by caregivers and children, its relationship with caregivers’ periodontal health-related quality of life, and described children’s dietary practices and oral hygiene habits. **Methods:** This cross-sectional study was conducted with caregivers of children participating in the Happy Smile Project in Birigui, São Paulo, Brazil. UPF consumption was assessed using a questionnaire based on the NOVA classification, and periodontal health-related quality of life was evaluated using the OHIP-14-PD. **Results:** A high frequency of UPF consumption was observed among both caregivers and children. Children whose caregivers had high UPF consumption were more likely to also present high consumption (OR = 9.96; 95% CI: 5.38–18.44; *p* < 0.001). Higher caregiver education was associated with lower odds of high UPF consumption among children. Children in the high-consumption group were older and showed higher consumption of sweetened milk beverages (*p* < 0.001). Risk behaviors for oral health, such as nighttime use of sweetened bottles and absence of toothbrushing afterward, were frequently reported. Regarding periodontal health-related quality of life, only the physical disability domain showed significantly higher scores among caregivers with high UPF consumption (*p* = 0.014). **Conclusions:** This study demonstrated that high consumption of ultra-processed foods by caregivers significantly increased the odds of children’s consumption and was associated with a greater negative impact on caregivers’ periodontal health-related quality of life, specifically in the physical disability domain. In addition, children exhibited a high frequency of oral health-damaging behaviors. These findings highlight the importance of family-centered strategies aimed at reducing the intake of ultra-processed foods and promoting healthier dietary and oral health behaviors.

## 1. Introduction

In recent decades, transformations in the global food system have led to a substantial rise in the consumption of ultra-processed foods (UPFs) [1]. According to the NOVA classification, these products are defined as industrials formulations composed predominantly of refined ingredients, additives, and substances intended exclusively for industrial use. They are characterized by high energy density, elevated concentrations of sugars, fats, and sodium, and limited nutritional value [2,3]. The growing consumption of UPFs has been consistently linked to adverse health outcomes, including metabolic disorders, chronic inflammatory conditions, obesity, diabetes, and rheumatoid arthritis [4,5,6]. Such associations have been observed across different age groups, affecting both adult and pediatric populations.

During childhood, the family environment plays a pivotal role in shaping dietary habits and oral hygiene behaviors, which directly influence the risk of developing oral diseases [7,8]. Parental practices such as role modeling, shared family meals, and responsive feeding strategies have been identified as key mechanisms in the development of children’s eating behaviors, highlighting the domestic environment as a determinant of healthier or less healthy dietary patterns [9].

Among oral diseases, dental caries and periodontal conditions remain among the most prevalent chronic disorders across the life course. Dental caries affect up to half of children under five years of age, whereas periodontitis impacts more than 60% of the adult population, with substantial consequences on quality of life and health systems [10,11,12]. Both conditions present a multifactorial etiology involving alterations in the dental biofilm as well as social and environmental determinants, and may progress to the destruction of dental and periodontal tissues when not adequately prevented or treated [13,14,15].

Recent evidence suggests an association between the consumption of ultra-processed foods (UPFs) and an increased risk of dental caries, particularly in childhood, as well as potential links with gingival and periodontal inflammatory processes in adults [4,16]. Common examples of ultra-processed foods include soft drinks, industrialized fruit juices, packaged snacks, chocolate-flavored beverages, sweetened dairy products, and ready-to-eat or frozen meals, which are widely consumed by both children and adults and are recognized for their potential negative impact on oral health [17,18]. Although it remains unclear whether these effects are amplified in family environments where UPFs are widely available and incorporated into daily routines, the domestic context has been recognized as a central determinant of children’s eating behavior [18].

However, several critical gaps remain in the literature. First, no studies have quantified the strength of association between parental and child UPF consumption using validated classification systems. Second, the mediating role of socioeconomic factors in this relationship remains unclear. Third, the impact of UPF consumption on periodontal health-related quality of life has not been investigated in populations with young children.

Despite growing interest in this topic, there is still a paucity of studies investigating, in an integrated manner, the relationship between parental or caregiver consumption of UPFs and its influence on children’s dietary intake. Furthermore, it is essential to understand the impact of high UPF consumption on adults’ periodontal health-related quality of life. Clarifying these associations is crucial to inform health promotion strategies that address common risk factors for oral and systemic diseases, particularly in interventions targeting early childhood. Therefore, this study had three primary objectives: (1) to quantify the association between caregiver and child UPF consumption patterns, (2) to examine the relationship between caregiver UPF consumption and periodontal health-related quality of life, and (3) to characterize children’s dietary practices and oral hygiene behaviors.

## 2. Materials and Methods

The study was approved by the Research Ethics Committee (CAAE: 89127725.0.0000.5420), in accordance with Resolution No. 466/2012 of the Brazilian National Health Council. All participants were informed about the objectives, procedures, risks, and benefits of the study and provided written informed consent. Anonymity, confidentiality of information, and the right to withdraw at any time without penalty were guaranteed.

**Figure 1:**
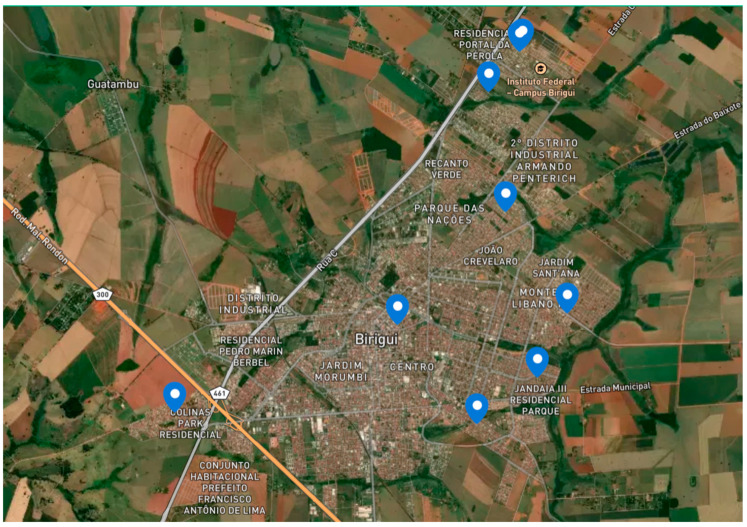
Geographic distribution of the participating schools on the map of Birigui—SP.

The Happy Smile Project is a university-based community outreach program developed by the School of Dentistry of Araçatuba (São Paulo State University, UNESP, Brazil) aimed at promoting oral health in early and preschool childhood. It involves the creation of a coordinated care network that brings together all groups responsible for the children’s health, development, and education. The program operates in public daycare centers and preschools, and its main activities include oral health education, the development of healthy eating and oral hygiene habits, as well as preventive and minimally invasive dental care within the school units. This cross-sectional observational study was conducted with legal caregivers of preschool children participating in the Happy Smile Project in the municipality of Birigui, São Paulo, Brazil. Schools were selected based on their geographic distribution across the city (Figure 1).

Sample size was calculated using the online calculator of the School of Dentistry of Bauru (University of São Paulo), considering a confidence level of 95%, a sampling error of 5%, and an estimated proportion of 20%, based on previous studies on ultra-processed food consumption in Brazil. A finite population of 4720 children enrolled in the Happy Smile project was considered. The minimum required sample size was estimated at 234 participants, with an additional 0.5% added for potential losses, totaling 235 individuals.

Legal caregivers of children within the established age range who agreed to participate and signed the informed consent form were included. Individuals who refused to participate or who did not adequately complete the questionnaires were excluded. For each analysis, participants with missing data were excluded pairwise.

Data collection was performed in person before parent–teacher meetings at the schools by a previously trained researcher.

### 2.1. Sociodemographic and Health Data

Caregivers provided sociodemographic information, including sex, age, education level, monthly household income (categories were standardized relative to the Brazilian minimum wage BRL 1518 per month in 2025), and number of residents in the household. Weight and height were self-reported for body mass index (BMI) calculation. Information on self-reported health conditions, such as arterial hypertension, diabetes mellitus, and hypercholesterolemia, was also collected.

### 2.2. Assessment of Ultra-Processed Food Consumption

Ultra-processed food (UPF) consumption was assessed using a structured questionnaire based on the NOVA classification and previous studies [3,19]. The instrument included 12 groups of UPFs: soft drinks; industrialized fruit juices (box or can); powdered drinks; chocolate-flavored beverages; flavored yogurts; packaged snacks or salted crackers; industrialized desserts (chocolate, ice cream, gelatin, or flan); processed meats (sausages, mortadella, ham); industrialized breads (sliced bread, hot dog or hamburger buns); industrialized sauces (mayonnaise, ketchup, or mustard); margarine; and ready-to-eat or semi-ready foods, such as instant noodles, packaged soups, and frozen meals. Participants reported whether each food group had been consumed in the 24 h prior to questionnaire administration, both for themselves and for their children. UPF consumption was categorized as high when five or more food groups were reported and low when fewer than five groups were consumed.

### 2.3. Children’s Dietary Practices, Oral Hygiene Habits, and Caregivers’ Perceptions

Information on children’s dietary and oral hygiene practices was collected using a questionnaire developed by the researchers. Data included nighttime bottle use, addition of sugar or chocolate powder to milk, and toothbrushing after bottle feeding. The questionnaire also included items on caregivers’ knowledge about ultra-processed foods, frequency of consumption, and perceived difficulties in offering a healthy diet and maintaining adequate oral hygiene for their children.

### 2.4. Periodontal Health-Related Quality of Life

The impact of caregivers’ periodontal condition on quality of life was assessed using the Brazilian validated version of the Oral Health Impact Profile for periodontal disease (OHIP-14-PD) [20]. The instrument consists of 14 items distributed across seven domains: functional limitation, physical pain, psychological discomfort, physical disability, psychological disability, social disability, and social disadvantage. Responses were recorded on a five-point Likert scale ranging from 0 (“never”) to 4 (“almost always”), with higher scores indicating greater negative impact. For analysis, item scores within each domain were summed, generating continuous quantitative domain scores.

### 2.5. Statistical Analysis

Data were analyzed using SPSS software, version 21.0 (SPSS Inc., Chicago, IL, USA), adopting a significance level of 5% (α = 0.05). Primary variables were ultra-processed food consumption by caregivers and children. Secondary variables included caregivers’ periodontal health-related quality of life and children’s oral health risk behaviors. Data normality was assessed using the Shapiro–Wilk test and visual inspection of histograms. Categorical variables were described using absolute and relative frequencies.

Associations between variables were evaluated using the Mann–Whitney U test and Spearman correlation coefficient. Binary logistic regression was performed to estimate odds ratios (ORs) and 95% confidence intervals (95% CI) for high UPF consumption among children, adjusted for caregiver education level, monthly household income, caregiver age, and child age.

## 3. Results

A total of 392 caregivers of children participating in the Happy Smile Project in Birigui, São Paulo, Brazil, were included and classified as low or high consumers of ultra-processed foods (UPFs). Participants with missing data were excluded from specific analyses as appropriate.

### 3.1. Caregivers’ Sociodemographic and Clinical Characteristics

Most caregivers were female (325/390), with similar distribution between low and high UPF consumption groups (*p* = 0.463). Regarding education (n = 376), most participants had completed high school (n = 231), followed by higher education (n = 73), elementary education (n = 51), and postgraduate studies (n = 21), with no significant differences between groups (*p* = 0.060). Monthly household income (n = 385) was most frequently between 1.5 and 5 minimum wages (n = 179), and no participants reported income above 12 minimum wages. Household size was predominantly three to four residents (n = 230), with no significant difference between consumption groups (*p* = 0.760). Mean age was 32.4 ± 7.2 years in the low-consumption group and 31.2 ± 7.3 years in the high-consumption group (*p* = 0.184). Regarding body mass index (BMI) (n = 312), most caregivers were classified as normal weight (n = 99) and overweight (n = 96), followed by obese class I (n = 72), followed by, with no significant differences between groups (*p* = 0.681). No significant associations were found between UPF consumption and self-reported hypertension (*p* = 0.955), diabetes mellitus (*p* = 0.353), or hypercholesterolemia (*p* = 0.743) (Table 1).

### 3.2. Children’s Sociodemographic and Behavioral Characteristics

Sex distribution did not differ between consumption groups (*p* = 0.838). Although the median age was identical in both groups (36 months), the age distributions differed significantly, with older children being more prevalent in the high-consumption group (*p* < 0.001). Nighttime bottle use was highly prevalent and not associated with UPF consumption (*p* = 0.893). In contrast, consumption of sweetened milk beverages (milk with sugar, chocolate powder, or similar) was significantly associated with higher UPF consumption (*p* = 0.0001), being more frequent in the high-consumption group. No significant association was observed between UPF consumption and toothbrushing after nighttime bottle use, although a borderline difference was detected (*p* = 0.058) (Table 2).

### 3.3. Frequency of UPF Intake

**Figure 2:**
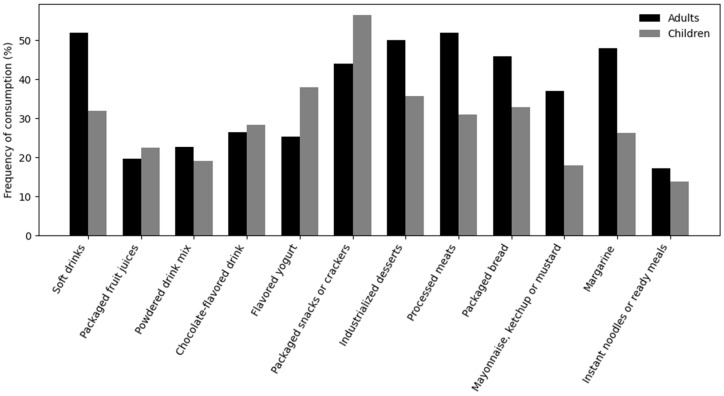
Frequency of consumption of ultra-processed food groups among adults and children.

All UPF groups showed significant correlation with overall UPF consumption among caregivers and children (*p* < 0.001), confirming consistency of the ultra-processed dietary pattern. Among caregivers, the most frequently consumed UPFs were soft drinks (52.2%), processed meats (51.9%), industrialized desserts (49.9%), and margarine (47.8%), followed by industrialized breads (45.8%) and packaged snacks (44.0%). Among children, the most frequently consumed UPFs were packaged snacks or salted crackers (56.4%), flavored yogurts (38.0%), industrialized desserts (35.7%), and industrialized breads (32.9%). Soft drinks were consumed by 31.9% of children and chocolate-flavored beverages by 28.3%. Instant noodles, packaged soups, and frozen ready-to-eat meals presented the lowest consumption frequencies (<17%) in both groups (Figure 2).

### 3.4. Association Between Caregivers’ and Children’s UPF Consumption

Table 3 presents the binary logistic regression analysis adjusted for caregiver education, household income, child age (in months), and caregiver age, including 342 participants. Children whose caregivers had high UPF consumption were significantly more likely to also present high consumption (OR = 9.96; 95% CI: 5.38–18.44; *p* < 0.001). Caregiver education level showed a protective effect: compared with the reference category, all higher educational levels were associated with lower odds of high UPF consumption among children (OR = 0.26; 95% CI: 0.10–0.64; *p* = 0.004; OR = 0.11; 95% CI: 0.03–0.34; *p* < 0.001; and OR = 0.14; 95% CI: 0.03–0.71; *p* = 0.017, respectively). Child age was positively associated with high UPF consumption, with each additional month increasing the odds by 3.9% (OR = 1.04; 95% CI: 1.02–1.06; *p* < 0.001). Household income was not significantly associated with children’s UPF consumption and remained in the model only as an adjustment variable (*p* = 0.840). Caregiver age was also not significantly associated with the outcome (OR = 0.98; 95% CI: 0.94–1.02; *p* = 0.268).

### 3.5. Reported Difficulties in Offering a Healthy Diet and Oral Hygiene

**Figure 3:**
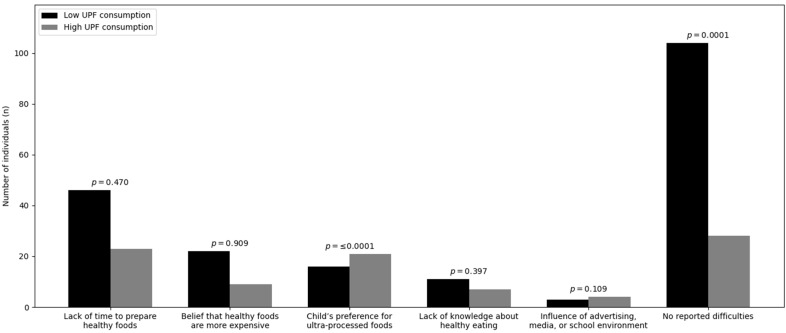
Difficulties in offering healthy food according to ultra-processed food consumption.

Lack of time to prepare healthy foods (*p* = 0.470), belief that healthy foods are more expensive (*p* = 0.909), lack of knowledge about healthy eating (*p* = 0.397), and influence of advertising or school environment (*p* = 0.109) were not associated with children’s UPF consumption Graph 3. In contrast, children’s preference for UPFs was significantly correlated with higher UPF consumption (*p* < 0.001) and was more frequently reported among caregivers of children in the high-consumption group Graph 3. The absence of reported difficulties in offering a healthy diet was more frequent among caregivers of children with low UPF consumption (*p* < 0.001) (Figure 3).

**Figure 4:**
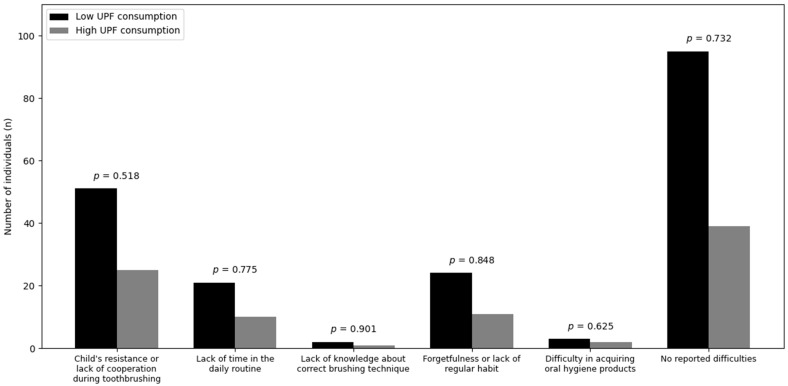
Difficulties in maintaining adequate toothbrushing according to ultra-processed food consumption.

Regarding oral hygiene, resistance or lack of cooperation during toothbrushing, lack of time, lack of knowledge of brushing technique, forgetting brushing, difficulty obtaining hygiene products, and absence of reported difficulties did not differ between low and high UPF consumption groups (*p* > 0.05 for all) (Figure 4).

### 3.6. Knowledge, Frequency, and Motives Related to UPF Consumption

Most caregivers (74.2%) reported knowing what UPFs are; however, 59.2% reported regular consumption. Practicality was the most frequently reported reason for consumption (36%), followed by personal preference (27.6%), taste (21.4%), and cost (13.5%). In addition, 25.3% reported never having reflected on the reasons for consuming UPFs.

### 3.7. Association Between UPF Consumption and Periodontal Health-Related Quality of Life

Scores of the OHIP-14-PD domains showed asymmetric distributions with predominance of low values and median scores equal to zero in most domains. No differences were observed between consumption groups for functional limitation, psychological discomfort, psychological disability, social disability, or social disadvantage (*p* > 0.05). Physical pain showed median values of 2.0 in both groups, with similar interquartile ranges (*p* > 0.05). Physical disability was the only domain that differed significantly between groups, with higher scores among caregivers with high UPF consumption (median 1 [IQR 4]) compared with low consumption (median 0 [IQR 3]) (*p* = 0.014) (Table 4).

## 4. Discussion

For the first time in the literature, the influence of parental or caregiver consumption of ultra-processed foods on children’s dietary intake has been systematically investigated. The study demonstrated a high frequency of ultra-processed food (UPF) consumption among both caregivers and children participating in the Happy Smile Project. Children whose caregivers presented high UPF consumption were almost ten times more likely to also consume these foods. These findings underscore the role of the family food environment in shaping children’s eating habits, indicating that caregivers’ dietary practices determine food availability and children’s consumption patterns, favoring the normalization of UPF intake.

The magnitude of the observed association suggests that UPF consumption in the household reflects a shared context of food choices, product availability, and family routines [21,22,23]. Previous studies confirm that children tend to reproduce dietary patterns observed in their caregivers, especially in early childhood, a period marked by dependence on parental decisions [9,18]. In addition, the contemporary food environment, including digital platforms and food delivery applications, has been described as strongly promoting UPF consumption, favoring its normalization within families. Factors such as wide availability, convenience, and lower relative cost contribute to high consumption, regardless of household income [24]. Daily childhood behaviors, such as longer screen time and reduced time spent in school settings, have been associated with lower consumption of fresh or minimally processed foods and higher intake of ultra-processed foods [25].

In this study, household income was not significantly associated with UPF consumption, although the literature reports heterogeneous findings between developed and developing countries. In high-income countries such as the United Kingdom, Canada, and the United States, higher UPF consumption has been associated with poorer socioeconomic conditions [26,27,28]. Conversely, in low- and middle-income countries such as Brazil and Colombia, studies indicate an inverse association, whereby populations with higher purchasing power show higher UPF consumption [29,30,31]. Thus, income alone is not a determining factor, and other aspects such as access and urbanicity may influence food choices [23].

Caregivers’ educational level showed a protective effect against UPF consumption, with higher education associated with lower odds of high consumption among children. However, national literature reports opposite trends, suggesting that as UPFs become more accessible, their consumption tends to spread across different educational strata [1,32].

A discrepancy between knowledge and UPF consumption was observed. This mismatch has been described in different contexts, indicating that knowledge alone is insufficient to modify eating behaviors and is counteracted by factors such as accessibility, preferences, and convenience [33,34]. Educational interventions without structural changes also show limited effectiveness, reinforcing that opportunity and motivation exert greater influence on food choices than acquired knowledge [35].

Although this study did not directly evaluate the prevalence of dental caries, certain risk behaviors—such as the nighttime use of sweetened bottles and the absence of subsequent toothbrushing—were identified and found to be associated with frequent consumption of ultra-processed foods (UPFs). Extensive evidence indicates that a higher intake of these products substantially increases the risk of early childhood caries [16,36]. Furthermore, family dietary patterns and caregivers’ oral health status may exacerbate this risk, underscoring the complex interrelationship between nutritional practices and oral health outcomes [37]. Sugar intake facilitates bacterial fermentation, resulting in acid production that promotes enamel demineralization. The frequent consumption of UPFs, particularly those rich in sugars and refined starches, renders these foods especially detrimental to oral health [36,37,38].

Increased UPF consumption was associated with older child age, in agreement with previous studies [39,40]. As children grow older, they gain greater dietary autonomy and increased exposure to environments outside the household, such as schools, social activities, and digital media, as well as greater influence from peers and food marketing strategies [41,42].

The investigation of the relationship between UPF consumption and periodontal health-related quality of life was innovative in this study and showed significant effects in the physical disability domain, such as gingival bleeding and chewing difficulties. Although inconsistent, evidence suggests that pro-inflammatory dietary patterns characterized by high intake of sugars and saturated fats are associated with increased risk of periodontal inflammation, whereas healthier dietary patterns exert a protective effect [43,44,45,46].

The selective association observed between ultra-processed food (UPF) consumption and the physical disability domain of the OHIP-14-PD requires careful interpretation. This domain encompasses functional limitations such as difficulty chewing and eating, which are more directly related to inflammatory changes in periodontal tissues than psychosocial dimensions. The absence of significant associations with the remaining OHIP-14-PD domains may be explained by several factors: (1) the relatively young age of the caregivers (mean 31–32 years), who are less likely to present advanced periodontal disease; (2) the cross-sectional nature of the study, which does not allow the assessment of cumulative or long-term effects; and (3) the possibility that UPF consumption influences periodontal health through biological pathways not fully captured by self-reported quality-of-life instruments. Future studies incorporating clinical periodontal examinations are warranted to corroborate these findings and better elucidate the underlying mechanisms.

This study has limitations that should be acknowledged. Its cross-sectional design does not allow causal inferences. Dietary intake was assessed using a single 24-h recall, which may not reflect habitual consumption and does not capture seasonal variation. Self-reported dietary and anthropometric data (weight and height) may be subject to recall and reporting biases, potentially underestimating ultra-processed food consumption.

Periodontal health and dental caries were not clinically assessed, limiting objective validation of oral health outcomes. Selection bias may be present, as participants were caregivers attending school meetings, possibly representing more health-conscious families. The study did not account for some potential confounders, such as food marketing exposure and neighborhood food environment. Generalizability is limited because data were collected in a single municipality. Finally, the application of the NOVA classification based on one-day recall and the use of pairwise deletion for missing data may have introduced bias.

These findings indicate the need for strategies to promote healthy eating that take into account the family, social, and economic environment. Extension projects such as the Happy Smile Project represent strategic settings for broader educational actions directed at caregivers and children, favoring healthier dietary practices. In this context, dentists occupy a strategic position in the promotion of comprehensive health; however, gaps still exist in dental education regarding the approach to dietary factors and common risk factors for oral and systemic diseases.

## 5. Conclusions

This study demonstrated that high consumption of ultra-processed foods by caregivers significantly increased the odds of children’s consumption and was associated with a greater negative impact on caregivers’ periodontal health-related quality of life, specifically in the physical disability domain. In addition, children exhibited a high frequency of oral health-damaging behaviors. These findings highlight the importance of family-centered strategies aimed at reducing the intake of ultra-processed foods and promoting healthier dietary and oral health behaviors.

## Figures and Tables

**Table 1:** Sociodemographic and clinical characteristics of caregivers according to the consumption of ultra-processed foods.

Demographic Data	Low Consumption of UPFs	High Consumption of UPFs	Total	Spearman’s Correlation (*p*-Value)
Gender (n = 390)				0.463
Female	179	146	325	
Male	39	25	64	
Not specified	1	0	1	
Education level (n = 376)				0.06
Elementary school	22	29	51	
High school	130	101	231	
Undergraduate degree	47	26	73	
Postgraduate degree	11	10	21	
Income (n = 385)				0.130
<1 minimum wage	16	19	35	
1–1.5 minimum wages	82	71	153	
1.5–5 minimum wages	105	74	179	
5–12 minimum wages	11	7	18	
>12 minimum wages	0	0	0	
N° of residents (n = 309)				0.760
Up to 2 people	19	19	38	
3–4 people	127	103	230	
5 people or more	22	19	41	
Age—mean (SD)(n = 353)	32.4 (7.2)	31.2 (7.3)	353	0.184
BMI (n = 312)				0.681
Underweight	4	1	5	
Normal weight	56	42	99	
Overweight	59	37	96	
Obesity class I	43	29	72	
Obesity class II	16	10	26	
Obesity class III	10	5	15	
Hypertension (n = 392)				0.955
No	192	152	344	
Yes	27	21	48	
Diabetes (n = 392)				0.353
No	201	163	364	
Yes	18	10	28	
Hypercholesterolemia (n = 392)				0.743
No	210	167	377	
Yes	9	6	15	

Data are presented as absolute values. Brazilian minimum wage = BRL 1518 per month (2025). The comparison between low and high ultra-processed food consumption groups was performed using Spearman’s correlation test.

**Table 2:** Sociodemographic and behavioral characteristics of children according to the consumption of ultra-processed foods (n = 392).

Demographic Data	Low Consumption of UPFs	High Consumption of UPFs	Total	Spearman’s Correlation (*p*-Value)
Gender (n = 392)				
Male	142	61	203	0.838
Female	134	55	189	
Age (months)—Median (IQR) (n = 351)	36 (25)	36 (24)	351	0.001
Nighttime bottle-feeding (n = 392)				
No	99	44	143	0.893
Yes	177	72	249	
Milk flavored with sugar, chocolate powder, or similar items (n = 392)				
No	176	43	219	0.0001
Yes	100	73	173	
Toothbrushing after nighttime bottle-feeding (n = 392)				
No	155	53	208	0.058
Yes	121	63	184	

Values expressed as n, median (IQR). Age comparison performed using the Mann–Whitney U test; associations evaluated by Spearman’s correlation coefficient. (α = 0.05).

**Table 3:** Association between caregivers’ consumption of ultra-processed foods and high consumption of these foods by children (n = 372).

Caregiver’s Consumption of Ultra-Processed Foods			
	OR	IC 95%	*p* value
Low Consumption	1.00	-	-
High Consumption	9.96	5.38–18.44	<0.001
Caregiver’s educational level			
	OR	IC 95%	*p* value
Elementary school	1.00	-	-
High school	0.257	0.103–0.644	0.004
Undergraduate degree	0.107	0.03–0.33	<0.001
Postgraduate degree	0.14	0.028–0.706	0.017
Family income			
	OR	IC 95%	*p* value
< 1 minimum wage	1.00	-	-
1–1.5 minimum wages	0.6	0.24–1.48	0.27
1.5–5 minimum wages	0.42	0.16–1.06	0.06
1.5–5 minimum wages	0.38	0.07–1.98	0.25
Children’s age (in months)			
	OR	IC 95%	*p* value
	1.039	1.018–1.061	<0.001
Caregiver age			
	OR	IC 95%	*p* value
	0.976	0.936–1.018	0.268

Binary logistic regression model adjusted for caregiver’s educational level and monthly family income. OR = odds ratio; 95% CI = 95% confidence interval. Income category > 12 minimum wages was not included in the model due to a lack of observations. *p*-values < 0.05 were considered statistically significant.

**Table 4:** Comparison of OHIP-14-PD domains according to caregivers’ consumption of ultra-processed foods.

Domain	Low UPFs Consumption	High UPFs Consumption	*p*-Value
Functional limitation	0 (0–2)	0 (0–2)	0.999
Physical pain	2 (1–4)	2 (1–4)	0.889
Psychological discomfort	0 (0–2)	0 (0–2)	0.174
Physical disability	0 (0–3)	1 (0–4)	0.014
Psychological disability	0 (0–2)	0 (0–3)	0.509
Social disability	0 (0–0)	0 (0–0)	0.107
Social disadvantage	0 (0–3)	0 (0–0)	0.275

Mann–Whitney U test. Values expressed as median and interquartile range.

## Data Availability

The dataset for this current consent is available from the corresponding authors upon reasonable request. Requests must justify the need for the data, ensuring it is for research purposes, while adhering to privacy, legal, or ethical restrictions that prevented immediate, open access.

## Abbreviations

The following abbreviations are used in this manuscript:

BMI	Body Mass Index
OHIP-14-PD	Oral Health Profile applied to Periodontal Diseases
OR	Odds Ratio
SPSS	Statistical Package for the Social Sciences
SD	Standard Deviation
UPF	Ultra-processed food
UPFs	Ultra-processed foods
